# Shape transformable bifurcated stents

**DOI:** 10.1038/s41598-018-32129-3

**Published:** 2018-09-17

**Authors:** Taeyoung Kim, Yong-Gu Lee

**Affiliations:** School of Mechanical Engineering, Gwangju Institute of Science and Technology, Gwangju, 61005 Republic of Korea

## Abstract

Non-invasive delivery of artificial implants, stents or devices in patients is vital for rapid and successful recovery. Unfortunately, because the delivery passage is often narrower than the size of the delivered object, a compromise between the shape that is effective at the targeted location and a thin form that allows smooth unobstructed travel to the destination is needed. We address this problem through two key technologies: 3D printing and shape memory polymers (SMPs). 3D printing can produce patient-customizable objects, and SMPs can change their initially formed shape to the final desired shape through external stimulation. Using these two technologies, we examine the design and fabrication of bifurcated stents. This study presents a mock-up where blood vessels are fabricated using moulded silicon, which supports the effectiveness of the proposed method. The experimental results reveal that a bifurcated stent with a kirigami structure can smoothly travel inside a vessel without being obstructed by branched parts. We believe that this work can improve the success rate of stent insertion operations in medicine.

## Introduction

The shape memory effects of materials have been known for a long time, and various applications of shape memory materials, such as fasteners, eyeglass frames, underwires for women’s brassieres, aircraft rivets, heat-shrinking tubes, and medical implants, have been widely recognized^1,2^. For a survey of example usages, we refer to two review papers on alloys^1^ and polymers^2^. Note that these applications utilize only simple geometries, such as disks, cylinders and arcs. We envision that more successful commercial applications will emerge through the use of complex geometries enabled by the use of advanced manufacturing processes such as 3D printing. Shape memory alloys (SMA)^3^ are still not very compatible with 3D printers, but an increasing number of shape memory polymers (SMPs) are currently being processed with 3D printers, giving rise to interesting applications. The application that we are presenting involves a branch of 3D printing that utilizes smart materials, including SMAs/SMPs. This application is called 4D printing. In 4D printing, the time-transient change in morphology is one of the key characteristics, and there are several materials, including SMAs and SMPs, that change their initially formed shape in response to external stimuli, such as temperature, humidity, electricity, and light. Compared to 3D printing, 4D printing requires careful considerations of the shape changes and possible side effects, such as self-collision, because of its dynamic nature^4–9^.

**Figure 1:**
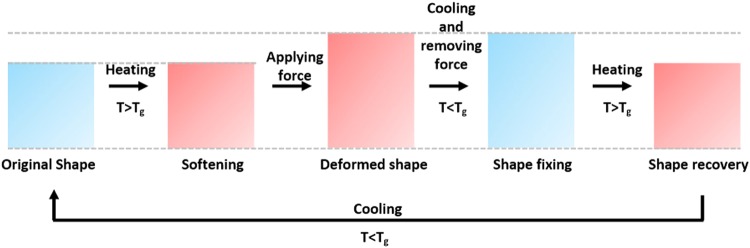
Shape fixing and recovery stages in shape memory polymers.

The processing of SMPs in 3D printing was once considered difficult. The highly viscous nature of shape memory thermoplastics often blocked the nozzles of extruders in fused deposition modelling (FDM)-based 3D printers. Moreover, shape memory thermosets were only available in private laboratories. The situation is changing because at present, we have several commercially available shape memory filaments. In addition, we are witnessing an explosion of published articles addressing SMPs in 3D printing^6–13^. SMPs can be initially transformed to a temporary shape via stimulation with heat or electricity with the addition of applied forces and subsequent cooling. The SMPs can later be returned to their original shape by further stimulation. Figure 1 illustrates the described material characteristics. The material in its original shape softens by heating it above the glass transition temperature (T_g_). When it is soft, we can easily change the shape by applying forces (similar to playing with dough), although the applied forces need to be sustained; otherwise, the material will return to its original shape. While keeping the applied forces intact, we cool down the object below its T_g_. Once cooled, the shape will be retained, even if we remove the applied forces. Surprisingly, if we heat the object above the T_g_ once again, the shape will deform back to its original state. This recovery of the initial shape is called the shape memory effect. SMPs possess the flexibility of polymers, and prior research efforts have achieved SMPs with biocompatibility and biodegradability, allowing them to be used for medical devices^14–16^. One of the most active applications in medicine is stents. In the human anatomy, blood vessels, lungs and biliary tracts are flow channels of important bodily fluids. Once these channels are blocked or narrowed, serious health problems can arise. Stents, often cylindrical in shape, are inserted into these congested locations to mechanically widen the pathways. Huang J. J. *et al*. have 3D printed a fistula stent using an FDM printer that uses a pharmaceutical grade thermoplastic polyurethane (TPU) filament. These researchers have also provided clinical results^17^. Although their work shows the successful usage of a 3D-printed patient-specific stent, we must emphasize that such practices are very rare and that no long-term follow-up study has been conducted. Using the same type of 3D printer, Robert van Lith *et al*. produced a stent using a copolyester material that can be biodegraded^18^. Furthermore, a cylindrical stent composed of a bioresorbable material was 3D printed using UV lamp photocuring^19^. Most stents used today are manufactured by knitting SMA wires using specialized frames. Due to the large demand for stents, they are mass-produced in several fixed shapes and forms. For a particular patient, the size that best matches the congested pathways is selected by the physician. Most pathways are cylindrical tubes; however, there are special needs for regions such as branches. In these areas, bifurcated stents are needed. We believe that the use of personalized stents can increase the chances of operation success, although such stents are not obtainable using modern production practices. The reason for this problem is that when SMA wire stents made in mesh structures are used, it is almost impossible to accommodate the size variations among the operating pathways. The problem is more severe for branched pathways because the shapes cannot be approximated with a single cylinder but rather require two cylinders with differing diameters. Bifurcated stents exhibit shapes that have significant protrusion on their sides, thereby making it extremely difficult to travel through the internal vessels to reach the target area. This difficulty is why, in practice, two separate stents are each delivered to the site and fixed at the operating location.

Kirigami comes from combining two Japanese words: ‘to cut,’ ‘kiri’, and ‘paper,’ ‘gami’. Kirigami is a superset of origami with the addition of cutting. Origami and kirigami start from a thin sheet that is folded (‘ori’) or cut (‘kiri’) according to special patterns, and by repeating these processes, a three-dimensional form can be obtained. Kirigami developed from art and is now being adopted in engineering^20–25^. For example, it has been used to study the movement of *Vorticella* and aortic heart valves in the area of biomimetic sciences^25–27^. Furthermore, the use of origami structure for stents has also been studied^28^.

In this study, we present a novel design and realization of a bifurcated stent through the use of kirigami structures and SMPs^29^. By utilizing kirigami structures, we were able to produce a transformable shape that can change from the initial compactly folded shape to ‘y’ shaped branched cylinders. The extremely challenging problem we have solved is how to collapse two upper branched tubes into a single tube. In this way, two bifurcated tubes were compacted to a sufficiently small size to pass through a tight cylindrical pathway. Furthermore, we thoroughly investigated the shape recovery of the proposed structure as a function of the applied temperature and the variation in stiffness based on the thickness and the number of repeated patterns in the kirigami structure. Finally, we demonstrated our idea through a mock-up where blood vessels were fabricated using moulded silicon. The physical bifurcated stents made by 3D printing with SMPs were later inserted into the artificial blood vessels and expanded to the desired shape, verifying the feasibility of our idea. We would like to emphasize that through 3D printing, we can obtain a patient-customized stent that will perfectly fit into the target branched vessels.

## Methods

### Design and Fabrication

3D modelling design was performed with NX (Siemens PLM software). Cylindrical and bifurcated stents were printed on a MakerPi 3D printer (M2030X, Shenzhen Soongon Technology Co., Ltd). The resolution of the printer is 0.05–0.30 mm. Filaments were extruded from an SMP pellet (MM-5520, SMP Technologies Inc.). The pellet consists of a polyurethane-based SMP. The stress-strain curve information about the pellet is downloadable from the manufacturer at the following location: http://www2.smptechno.com/en/smp/post_15.html. The printing bed was not heated, and the nozzle temperature was raised to 210 °C. The blood vessel mock-up was made by solidifying silicon (KE-1606, ShinEtsu) mixed with a hardener at room temperature with 3D-printed blood vessels placed at the centre. The blood vessel mock-up was later finalized by cutting the solidified silicon and carefully extracting the 3D-printed core.

### Analysis

The images and videos were obtained from a digital camcorder (HDR-CX450, Sony). The recorded footage was analysed by motion analysis software (ProAnalyst, Xcitex). Fiducial markers were placed on the specimen to record the position data that were used to compute the distances and radii of curvatures. Compression test simulations were performed on finite element analysis (FEA) software (ANSYS, ANSYS Inc.) (see SI for details).

## Results

### Single cylindrical tube made from a kirigami structure

**Figure 2:**
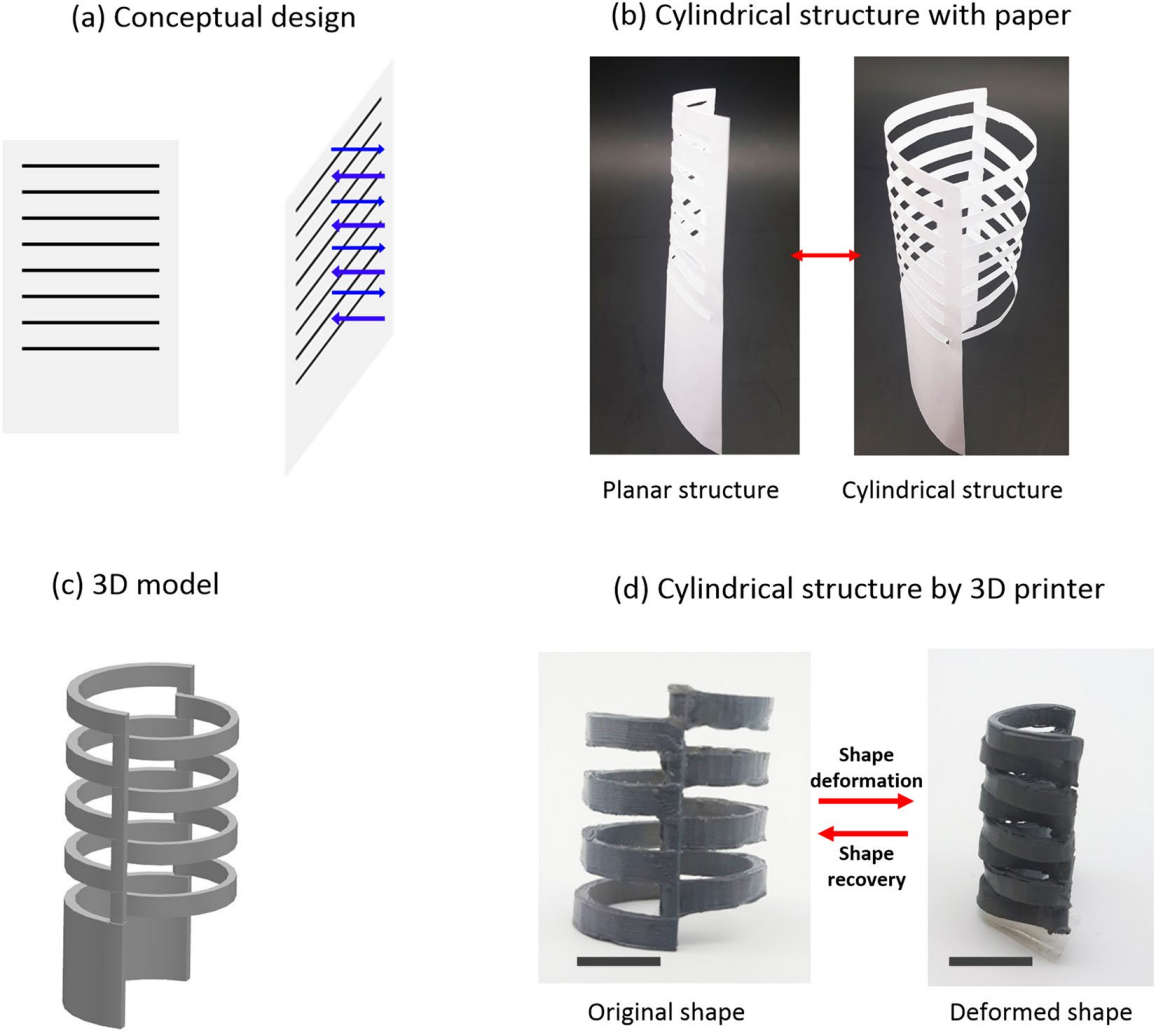
Design process of a tube. (**a**) The black lines denote cutting lines, and the blue arrow lines denote loading directions. (**b**) Working example of the conceptual design with paper. (**c**) A digital 3D model. (**d**) A 3D printed tube and its compacted state. The scale bar is 10 mm. The stents were post-processed with black paint for visual clarity in (**d**).

We first illustrate the design and fabrication of a kirigami-based single cylindrical tube representing a stent. The most important design criterion for a stent is that the shape should be tubular such that blood and bodily fluids can flow without obstruction through the inner pathway. Figure 2(a) illustrates our conceptual design of the kirigami structure. In this figure, the black lines denote cutting lines, and the blue arrow lines are directions and locations of the forces that will be applied to expand the shape. Note the interchanging directions of the applied forces in the vertical direction. The physical realization of these concepts is depicted in Fig. 2(b). Note that the left curled sheet of paper is expanded to a cage-like cylinder to the right. This idea is later used to fabricate 3D-printed tubes. The modelling was performed in a commercial 3D modeller, as shown in Fig. 2(c). The 3D model was later used to 3D print the stent, as shown on the left side of Fig. 2(d). The printed stent was subsequently softened by immersing the object inside a water basin where the temperature was set slightly above the T_g_. After softening, the stent was removed in order to shape it into a compact structure (a half cylinder) and immediately cooled down while applying pressure so that the changed shape was retained during cooling. Once cooled, the shape is memorized, and by heating it above the T_g_, it will recover its memorized shape.

**Figure 3:**
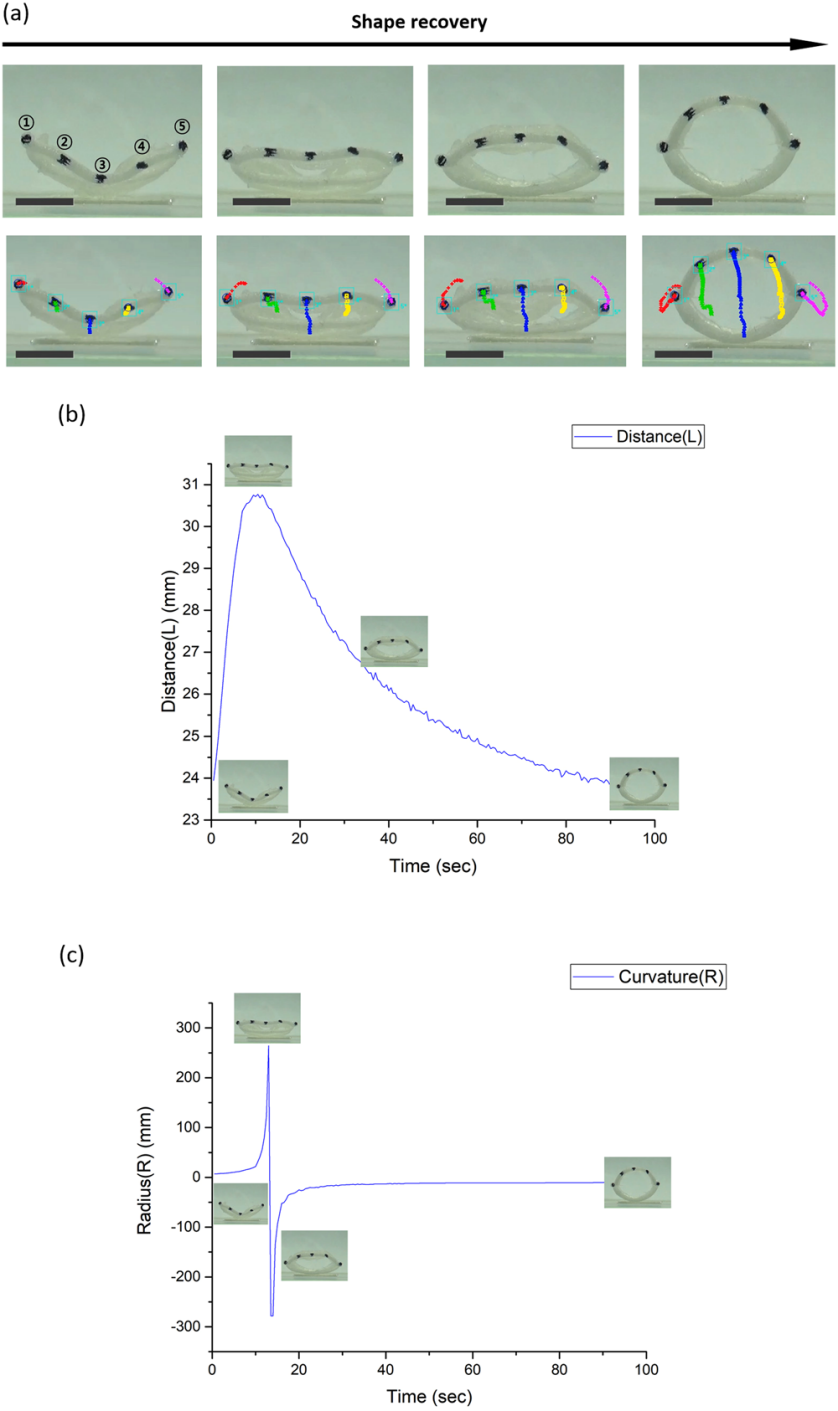
Shape recovery image analysis of a tube at 50 °C. (**a**) Five fiducial tracking points and their trajectory during shape recovery. (**b**) Distance plot of ① and ⑤ as a function of time. (**c**) Radius of curvature plot as a function of time. Positive values denote bulging towards the bottom. The scale bar for (**a**) is 10 mm.

**Figure 4:**
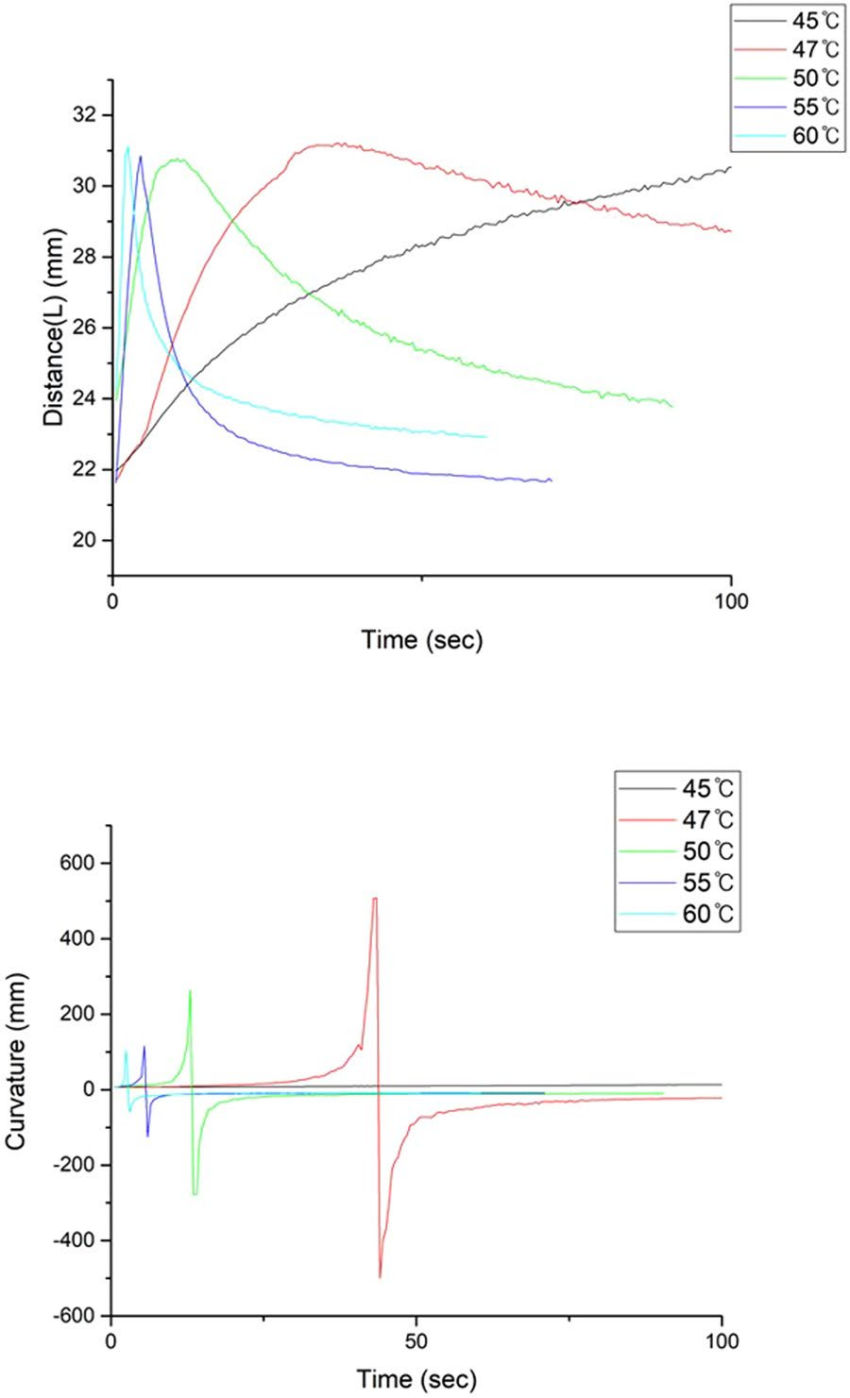
Recovery characteristics at various ambient temperatures.

To quantify the recoverability of the cylindrical tube, we put fiducial markers along the top of the cylinder and tracked their trajectory by using image analysis software. The analysis was performed at 50 °C, and Fig. 3 shows the recovery results. Figure 3(b) plots the distance between points ① and ⑤ in Fig. 3(a). The peak value in this graph shows the moment when the curvature sign of the upper half of the cylinder is reversed. This moment is also when the upper half suddenly snaps outward from the containing lower half of the cylinder. The distance reaches its original value once unfolding concludes. Figure 3(c) plots the radius of curvature at point ③, approximately obtained by circumscribing a circle around three points: ②, ③, ④. Notice that the sign of the curvature changes at 13 secs, which denotes that the bulge that was directed downwards moved to the upward direction after snapping through. The recovery rate of an SMP through heat depends on the applied temperature. We performed additional experiments to determine the effect of temperature on the recovery rate, as shown in Fig. 4. Figure 4(a) plots the distance between points ① and ⑤ in Fig. 3(a), again with various temperature settings. The time when the structure reaches its peak point shortens as we increase the applied temperature. Similarly, as shown in Fig. 4(b), the moment in which the sign of the radius of curvature is reversed occurs more quickly as we increase the applied temperature.

### Finite element analysis simulations

**Figure 5:**
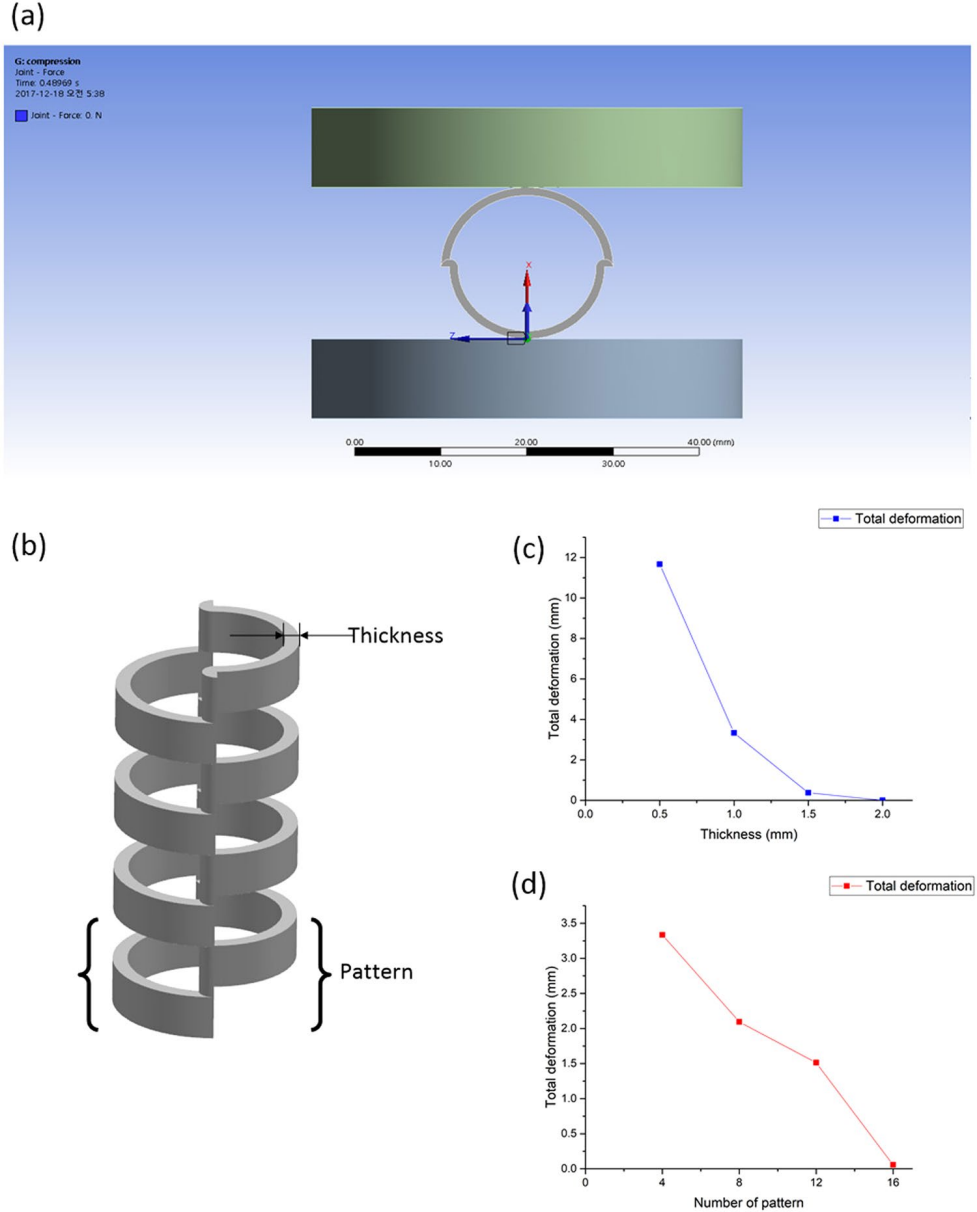
Compression simulation of a cylinder shape. (**a**) Upper plate (fixed support) and lower plate (force applied). (**b**) Definition of thickness and pattern. (**c**) 10 N compression test result with various thicknesses. (**d**) 10 N compression test results with various numbers of patterns.

We investigated how the shell thickness and the number of repeated patterns affected the stiffness of the structure. The investigation was conducted through finite element analysis (FEA) simulation. The stiffness is important because pathways cleared by the stents can undergo compression and because we want the structure to withstand the external disturbances, not to collapse. We followed the analysis performed by Han *et al*.^30^ FEA simulation was carried out with commercial software (ANSYS). The model variation was performed by changing the thickness and the number of patterns, as shown in Fig. 5(b). The ambient temperature was set at room temperature (25 °C). In this simulation, we did not consider the thermal effect. As illustrated in Fig. 5(a), the cylinder was compressed by fixing the upper plate and raising the lower plate upward at 10 N (see SI for details). For the reference model that was used in this article, with a 1 mm thickness and 4 patterns, a compression of 10 N resulted in 17.54% compression. For other experiments with various thicknesses and numbers of patterns, we were able to conclude that the deformation decreases (increase in stiffness) for thicker tubes and for tubes with a greater number of patterns. The results are shown in Fig. 5(c,d). Detailed material properties are given in Table S2. The numerical results showed an inverse relation between the deformation and the thickness of the stent, as well as between the deformation and the number of patterns. The results shown in this study can be used to determine how the designer can modify the stent to achieve the same goal, i.e., structural stiffness versus external compressive forces.

### Bifurcated stent using kirigami structure

**Figure 6:**
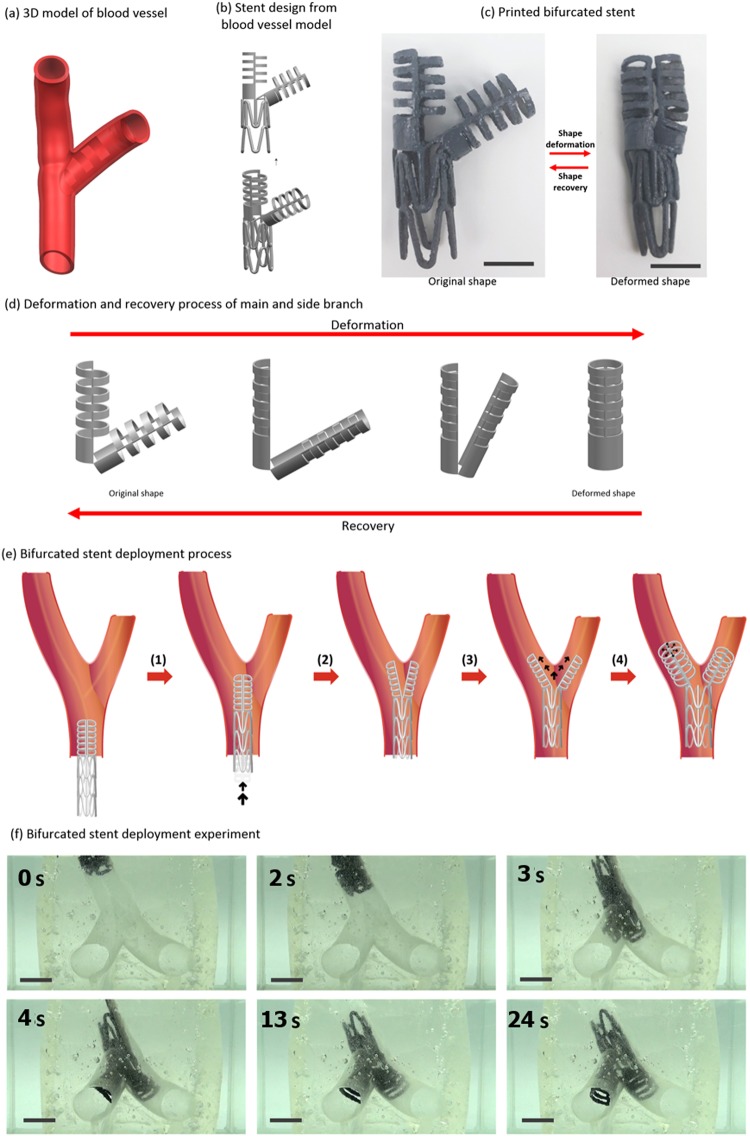
Bifurcated stent design and deployment. The scale bars for (**c**) and (**f**) are 20 mm. The stents were post-processed with black paint for visual clarity in (**c**) and (**f**). The length and width of the folded struts shown in (**c**) are 80 mm and 50 mm, respectively.

Based on the cylindrical tube using kirigami structures, we designed a bifurcated stent. The stent was designed such that it would fit snugly inside the target branched blood vessels shown in Fig. 6(a). The outer diameter of the stents is equal to the inner diameter of the vessels, which is approximately 22 mm. The design of the lower trunk (Fig. 6(b)) of the stent was borrowed from conventional designs where circumferential contractions and expansions were known to be effective. Our kirigami design shown in Fig. 6(b) is applied starting from the junction up to the branched tubes. The digital design was sent to a 3D printer and printed as shown on the left of Fig. 6(c), and the compacted form is shown on the right. The detailed stages of the transformation from the compacted shape to the deployed shape are shown in Fig. 6(d). We attached two of the previously described tubular kirigami structures that were folded into half cylinders and closed by touching them other, similar to an alligator mouth. Once closed, the structure appears to be one cylinder. We will explain this in more detail. In Fig. 2(d), the right half of the cylinder is first collapsed and nested into the left half cylinder. Subsequently, two concentric half cylinders can be further compacted by increasing the curvature. This secondary compaction enables the stent to consume less space than the completely unfolded diameter. The closed and compacted stent enters the tunnel as shown in Fig. 6(e). To measure the Tg, we conducted a thermomechanical analysis of the 3D-printed specimen. The result was that 50 °C was the optimal temperature. As we reached the target location, we raised the ambient temperature above the Tg. We raised the temperature of water by mixing 1500 ml of water heated at 90 °C with 2000 ml of 25 °C water. The equilibrium temperature measured with a digital thermometer was approximately 51 °C. The raised temperature will open and expand the branching tubes that will be further guided by themselves and enter the branch vessels. After a period of time, the expansion finalizes, and the stent perfectly fits the branched vessels. Figure 6(f) shows a mock-up experiment of the whole process. The branched vessels were made by using a 3D-printed blood vessel as a mould. Liquid silicon was solidified with the 3D-printed blood vessel held inside and subsequently removed. We can see that the insertion and expansion stages were performed as prescribed, and the time shots at 7 and 8 sec show the important moments when the two branches separate and each enters its corresponding tunnel. The two branches were folded together as they were inserted into the bifurcating site. The end tip of the folded dual branches starts to divide due to the heating of the stent by the surrounding medium. The division is further expedited by the operator pushing as each branch is inserted into its target vessel. After full entry, the cylindrical branch tubes expand to their full size, and the deployment is completed.

## Discussion

In this work, we presented a novel design and fabrication of a bifurcated stent that utilizes kirigami structures. Kirigami structures allow the shape to change between a thin sheet and a volumetric object. The 3D-printed kirigami bifurcated stent was folded into a compact shape that allowed it to travel through the vessel without interfering with the inner vessel walls. This compact shape resembles a smooth cylindrical pipe and can smoothly travel inside the vessel without being obstructed or hindered. Upon reaching the target location, the stent was triggered to return to its expanded form for the purpose of expanding the blocked or narrowed blood vessels. Through FEA simulations, we were able to find the relation between the thickness and the number of patterns in terms of their effect on the stiffness of the kirigami structure. For this kirigami structure to be clinically used, the stiffness should be tailored to the particular deployed site. For example, the stents should be stiffer when deployed in an artery than in the bile duct. Furthermore, the force-displacement behaviour and critical force of the collapse, as well as the experimental result, should all be thoroughly investigated. For the purpose of maintaining the integrity of the blood vessel, a stiffer stent is necessary. Finally, we fabricated our design using an FDM-based 3D printer with SMP filaments. The use of 3D printers enabled us to develop stents that are exactly the negative replica of the blood vessel. This method will greatly increase the effectiveness of the proposed stent in areas where there are highly irregular forms of blood vessels. Although we have not addressed the issues of the biocompatibility, biodegradability and high Tg of our material, these problems can be solved by referring to previous works that have reported biocompatible and biodegradable SMP materials^14,31,32^.

### Limitations of the Study

The novel kirigami-based bifurcated stent structure that we have proposed provides a new approach where shape transformation can greatly ease the problems of the hindrance and obstruction of conventional stent structures when used in bifurcating vessels. However, there still exist many unsolved and difficult problems to be overcome before the proposed method can actually be used for patients. First, the SMP used for the demonstrated kirigami structure was not tested for biocompatibility. Second, the shape transformation temperature of the demonstrated material is too high for clinical use. Although previous works^14^ have shown the existence of biocompatible SMPs, extensive tests need to be performed to clinically use our method. Third, the stents are too thick to be clinically used. This thickness was the thinnest structure that we could produce using the material and the printer that were utilize for the experiment. For actual clinical applications, much thinner and smaller stents must be developed, along with a material that is biocompatible, as noted above. Finally, we need to measure the radial forces of the proposed structure in real patient situations. We need to verify whether the exercisable radial force is strong enough to expand vessels obstructed with plaque. Therefore, the contribution of the proposed method needs to be assessed on structural shape changes that are very well suited for bifurcating vessels. Indeed, many studies on force measurements, biocompatibility, and clinical verification need to be done in order for patients to actually benefit from the proposed method.

## Electronic supplementary material

Supplementary Information
