# Supplementary material for: Shape transformable bifurcated stents
Source: Sci Rep. 2018 Sep 17;8:13911. doi: 10.1038/s41598-018-32129-3 (PMC6141457; doi:10.1038/s41598-018-32129-3)
Supplement: Supplementary file 1 — Supplementary Information [file 41598_2018_32129_MOESM1_ESM.pdf]

Supplementary information

Shape transformable bifurcated stents

Taeyoung Kim, Yong-Gu Lee*

All authors are with School of Mechanical Engineering, Gwangju Institute of Science and Technology, Gwangju, Republic of Korea.

* Contact information of corresponding authors:

Yong-Gu Lee(email : lygu@gist.ac.kr.)

Figure Captions

Supplementary Table S1 The measured elastic modulus of shape memory polymer filament using thermomechanical analysis(TMA Q400EM, TA Instruments).

Supplementary Table S2 The properties of shape memory polymer filament from SMP Technologies Inc.

Supplementary Figure S3 The height and diameter of a cylindrical tube

Supplementary Figure S4 The simulation result of 10N compression applied to a cylinder with varied thickness and number of patterns. (a) the result of thickness changes(n=4 fixed). (b) the result of number of pattern changes(t = 1mm fixed)

- Finite element analysis condition

We have used static structural module provided by ANSYS(FEA tool) workbench. The standard dimensions of the cylindrical tubes are as follows; height = 40mm, diameter = 20mm (Fig S3) mesh size= 1mm. In Fig S4, the upper plate was set as a fixed support and the lower plate was used to apply compressive forces to the structure. Material properties are shown in Table S1 and S2.

Table S1 The measured elastic modulus of shape memory polymer filament using thermomechanical analysis(TMA Q400EM, TA Instruments).

Temperature(℃)	Elastic modulus(MPa)
22	444.4
25	403.74
27	257.6
35	141.2
45	33.38
55	5.98
65	2.84
75	0.67

Table S2 The properties of shape memory polymer filament from SMP Technologies Inc.

Density	1.21g/cm^3
Glass transition temperature	55℃
Poisson ratio	Below T_g : 0.40<br>Above T_g : 0.45
Tensile yield strength	48MPa

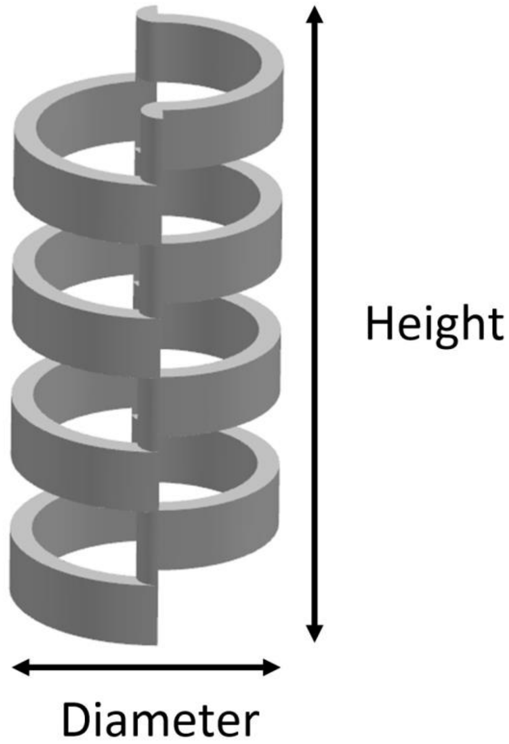

Figure S3 The height and diameter of the cylindrical tube

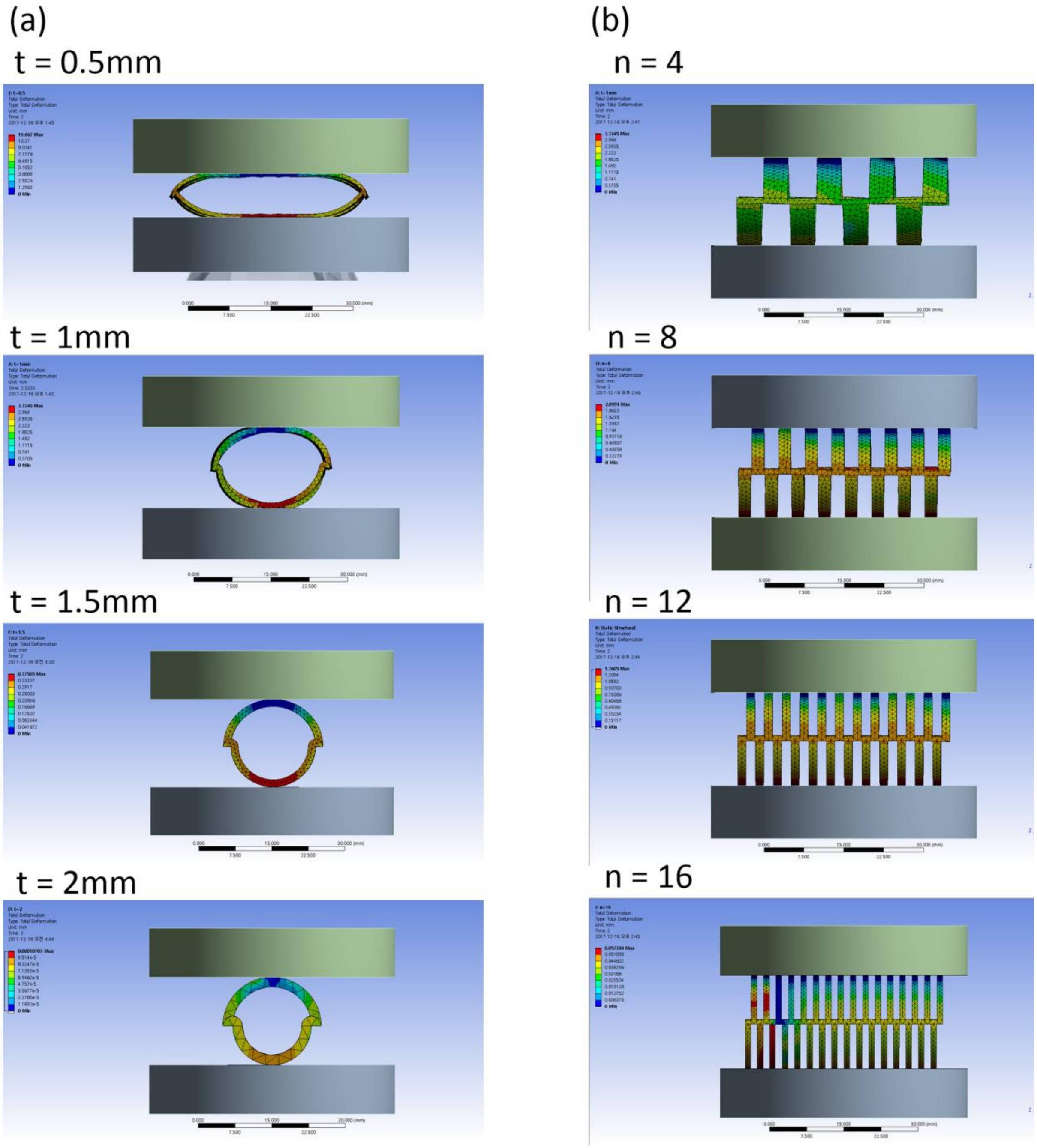

Figure S4 The simulation results of 10N compression applied to a cylindrical tube with varying thickness and number of patterns. (a) the result of thickness changes(n=4 fixed). (b) the result of number of pattern changes(t = 1mm fixed)
